# 1315. Ceftaroline Versus Vancomycin for the Treatment of Acute Pulmonary Exacerbations of Cystic Fibrosis in Adults

**DOI:** 10.1093/ofid/ofab466.1507

**Published:** 2021-12-04

**Authors:** Marc Esquivel, Marguerite Monogue, Greg Smith, James D Finklea, James Sanders

**Affiliations:** 1 UT Southwestern Medical Center, Dallas, Texas; 2 University of Texas Southwestern Medical Center, Dallas, Texas; 3 University of Texas Southwestern, Dallas, TX

## Abstract

**Background:**

Methicillin-resistant *Staphylococcus aureus* (MRSA) is a prominent colonizer in cystic fibrosis (CF) patients that causes acute pulmonary exacerbation (APE). Vancomycin is the first line treatment for APE of CF; however, optimal alternatives remain poorly defined. The goal of this study was to determine the safety and efficacy of ceftaroline in CF patients presenting with an APE caused by MRSA.

**Methods:**

This study was a single-center, retrospective cohort study from January 1, 2011 to January 1, 2020. The study included adult CF patients admitted for APE with %FEV1 > 10% lower than the patient’s baseline. A positive MRSA culture within 90 days before or 21 days after hospital admission and receipt of > 7 days of either vancomycin or ceftaroline was required for inclusion. Patients were excluded for receipt of a lung transplant, > 48 hours of alternative MRSA therapy, renal replacement therapy, or an APE secondary to fungal or mycobacterium infection. The primary outcome was the return to > 90% of baseline lung function measured by discharge %FEV1 in comparison to baseline %FEV1.

**Results:**

Fifty-six patients were included in the analysis (22 ceftaroline; 34 vancomycin). There were no differences in baseline characteristics (Table 1). Eleven (50%) patients in the ceftaroline group and 19 (56%) in the vancomycin group met the primary outcome (P = 0.79) (Figure 1A). FEV1 measurements at baseline, admission, and discharge were not different between treatments (Figure 1B). Patients treated with ceftaroline had a longer length of stay during hospital admission, 14 days (IQR 13-14) vs.10 days (IQR 7-14), P = 0.01. Other secondary outcomes were similar between the ceftaroline and vancomycin groups, respectfully, including 30-day readmission rate, 6 (27%) vs. 12 (35%), P = 0.57; 30-day mortality, 0 (0%) vs. 2 (6%), P = 0.51; neutropenia 3 (12%) vs. 1 (3%), P = 0.29; *Clostridioides difficile* infection 0 (0%) vs. 1 (3%), P = >0.99; or acute kidney injury 2 (9%) vs. 5 (15%), P = 0.69.

Table 1. Baseline characteristics for ceftaroline and vancomycin treated patients

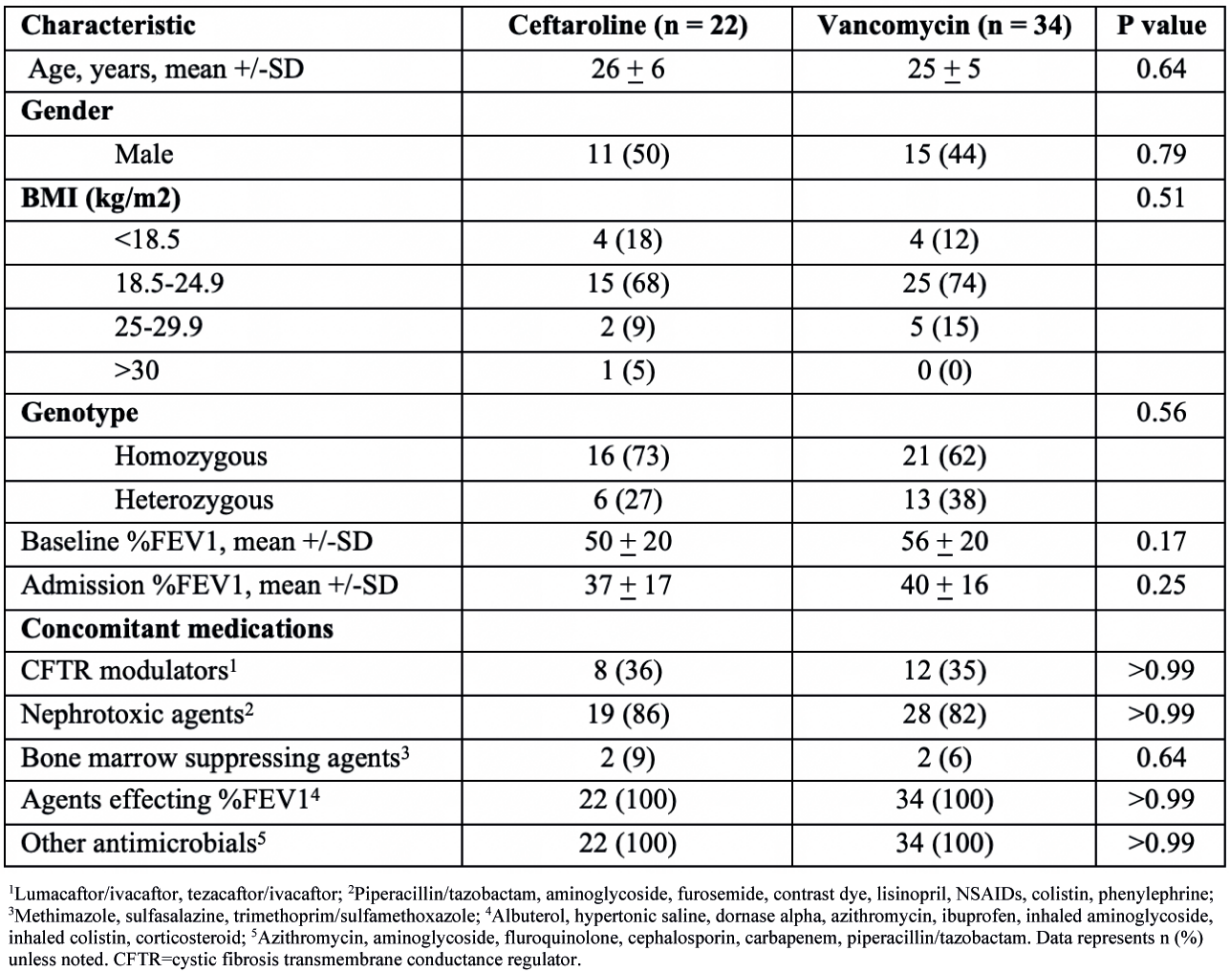

1Lumacaftor/ivacaftor, tezacaftor/ivacaftor; 2Piperacillin/tazobactam, aminoglycoside, furosemide, contrast dye, lisinopril, NSAIDs, colistin, phenylephrine; 3Methimazole, sulfasalazine, trimethoprim/sulfamethoxazole; 4Albuterol, hypertonic saline, dornase alpha, azithromycin, ibuprofen, inhaled aminoglycoside, inhaled colistin, corticosteroid; 5Azithromycin, aminoglycoside, fluroquinolone, cephalosporin, carbapenem, piperacillin/tazobactam. Data represents n (%) unless noted. CFTR=cystic fibrosis transmembrane conductance regulator.

Figure 1. %FEV1 trend from baseline to discharge in patients treated with ceftaroline or vancomycin

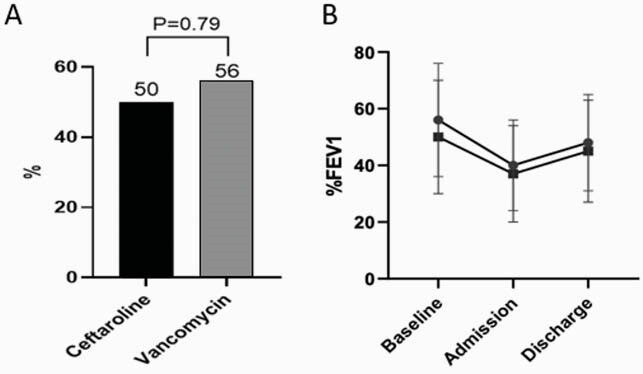

(A) Percentage (%) of patients who met the primary outcome in each group; (B) Mean %FEV1 change between ceftaroline (square) and vancomycin (circle) with error bars representing standard deviations

**Conclusion:**

This study found no difference in safety and efficacy outcomes between vancomycin and ceftaroline. Our small cohort supports ceftaroline as an alternative agent for the treatment of MRSA mediated APE of CF.

**Disclosures:**

**All Authors**: No reported disclosures

